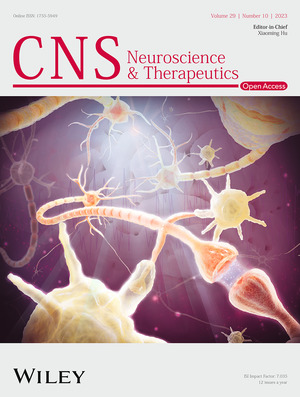# Front cover

**DOI:** 10.1111/cns.14467

**Published:** 2023-09-11

## Abstract

The cover image is based on the Original Article *Preferential pruning of inhibitory synapses by microglia contributes to alteration of the balance between excitatory and inhibitory synapses in the hippocampus in temporal lobe epilepsy* by Jianchen Fan et al., https://doi.org/10.1111/cns.14224.